# FLIFuse: a lightweight parallel multi-branch network for pneumonia pathogen identification in chest x-rays

**DOI:** 10.3389/fcimb.2026.1938619

**Published:** 2026-10-27

**Authors:** Yi Yang, Zhaoli Yao, Zhikun Zhang, Meifang Yu, Chong Yao, Liangquan Jia, Feng Hua

**Affiliations:** 1 Huzhou Central Hospital, Affiliated Central Hospital of Huzhou Normal University, Huzhou, China; 2 School of Information Engineering, Huzhou Normal University, Huzhou, China; 3 Jinhua Guangfu Oncology Hospital, Jinhua, China

**Keywords:** chest X-ray, computer-aided diagnosis, lightweight multi-branch network, multi-receptive field feature fusion, pneumonia pathogen identification

## Abstract

**Introduction:**

Pneumonia is one of the deadliest lower respiratory tract infections worldwide, and rapidly distinguishing bacterial pneumonia, viral pneumonia, and normal chest radiographs is crucial for guiding precise medication and avoiding misdiagnosis. However, the imaging manifestations of bacterial and viral pneumonia on chest X-rays often overlap, and some normal chest X-rays may also show atypical changes. Clinical differentiation relies heavily on the expertise of senior physicians. In primary care or emergency settings where experienced radiologists are scarce, this can readily lead to diagnostic delays and antibiotic misuse, further exacerbating the risk of antimicrobial resistance and severe outcomes.

**Methods:**

To address this challenge, this study proposes a lightweight network named FLIFuse. By adopting a parallel multi-branch architecture, the model thoroughly mines discriminative imaging features, substantially reducing computational overhead while maintaining high classification accuracy. This provides a technical solution that balances accuracy and efficiency for medical image analysis in resource-constrained environments. FLIFuse consists of three heterogeneous branches: an ExtraDW module that enhances local texture details, a Conv2Next module that captures global semantic structures, and an FLI Block that fuses multi-granularity information across different receptive fields to strengthen the response in critical regions.

**Results:**

The model occupies only 33.43 M parameters and incurs a computational cost of 6.61 GFLOPs. On the public ChestXRay2017 dataset, FLIFuse achieves a three-class classification accuracy of 91.4% for normal, bacterial pneumonia, and viral pneumonia, outperforming several current state-of-the-art models and attaining extremely low resource consumption while ensuring diagnostic accuracy. In cross-domain tests on an external COVID-19 chest X-ray dataset and the Nerthus endoscopic image dataset, the model also exhibits stable transferability, demonstrating its potential for cross-domain adaptation across different medical imaging tasks.

**Discussion:**

These results demonstrate that FLIFuse strikes a favorable balance between diagnostic accuracy and computational efficiency, providing an efficient and lightweight model option for computer-aided interpretation of pneumonia imaging, and showing potential for translation into clinical auxiliary diagnosis.

## 1 Introduction

Pneumonia is one of the respiratory diseases with the highest mortality rates worldwide. Although bacterial and viral pneumonia exhibit highly similar early clinical manifestations, they differ fundamentally in terms of treatment regimens, prognosis, and risk of drug resistance; accurate differentiation is key to optimizing treatment and improving prognosis (Bender et al., 2024). However, current clinical diagnosis which primarily relies on traditional methods such as clinical symptoms, laboratory tests, and imaging analysis—faces numerous challenges (Metlay et al., 2019). On the one hand, the high degree of overlap in the clinical manifestations of bacterial and viral pneumonia increases the risk of misdiagnosis, and the accuracy of existing biomarkers for differentiation is limited (Huang et al., 2026; Yu and Tang, 2026). On the other hand, laboratory testing methods suffer from limitations such as long turnaround times, low positive rates, and susceptibility to contamination; moreover, their accuracy is highly dependent on the timing of sampling and pathogen load (Musher and Thorner, 2014; Swaminathan et al., 2026). Furthermore, imaging features also overlap, and their interpretation relies heavily on the physician’s experience, making it highly subjective and inefficient—a problem further exacerbated by a shortage of specialized radiologists, particularly in primary care facilities (Franquet, 2018).

In recent years, with the rapid development of artificial intelligence and deep learning technologies, convolutional neural networks (CNNs) have become a core technology in medical image analysis, particularly in the field of pneumonia diagnosis. CNNs can automatically extract features from large volumes of image data, improving diagnostic efficiency and accuracy, and have demonstrated exceptional capabilities, especially in identifying subtle lesions and atypical cases (Litjens et al., 2017; Esteva et al., 2021). For example, Apostolopoulos et al. used transfer learning on a VGG19 network model to perform binary classification of chest X-ray images, achieving an accuracy of 98.75%, and a three-class classification accuracy of 93.48% (Apostolopoulos and Mpesiana, 2020). Recent studies have made further significant progress: Akrout et al. proposed a decision-level fusion model based on transfer learning that combines ResNet50 and ResNet101, achieving an accuracy of 95.68% on chest X-ray pneumonia classification and 99.61% on CT images (Akrout et al., 2026); Muthukumaraswamy et al. proposed the CE-Cascade framework, which combines CNNs with cascaded forests and achieved an overall classification accuracy of 98.86% on a multimodal dataset containing bacterial, viral, and fungal pneumonia (Muthukumaraswamy et al., 2026); Huang et al. proposed an attention-enhanced ResNet50V2-SE, which achieved an accuracy of 98.18% in the four-class classification of pneumonia, namely normal, bacterial, viral, and COVID-19, demonstrating the effectiveness of attention mechanisms in enhancing the diagnostic performance of CNNs (Huang et al., 2025).In addition, some studies have conducted systematic analyses from the perspectives of model comparison and clinical deployment. Rahman et al. 69 fine-tuned four models, including Alex Net and ResNet18, achieving a binary classification accuracy of 98% and a three-class accuracy of 93.3%, verifying that transfer learning can effectively alleviate data scarcity Rahman et al. (2020). Jain et al. compared a self-built CNN with pretrained networks such as VGG16 and ResNet50. and found that the self-built three-layer CNN achieved an accuracy of 92.31%, outperforming models like VGG16 (Jain et al., 2020). Shakeri and Far compared three transfer learning architectures DenseNet121, DenseNet169. and ResNetlOD and found that DenseNet121 achieved the highest validation accuracy of 98.68% Shakeri and Far (2024) Nevertheless, due to the high overlap of visual features across different categories in medical images and significant intra-class variations, relying solely on local features captured by CNNs often fails to adequately model global semantic information, which largely limits further improvements in classification performance.

The Transformer architecture (Vaswani et al., 2023) was first proposed by Vaswani et al. in 2017. It initially revolutionized the field of natural language processing and was subsequently extended to computer vision, giving rise to the Vision Transformer (ViT) (Dosovitskiy et al., 2021). By dividing images into patches and utilizing self-attention mechanisms to extract global information, ViT demonstrates powerful feature modeling capabilities in medical image classification tasks. However, ViT still suffers from high computational complexity, difficulties in processing high-resolution medical images, and insufficient modeling of local features. To address these issues, the Swin Transformer proposed by Liu et al. introduces a sliding window mechanism that computes self-attention within local windows and facilitates cross-region information exchange through window shifting, thereby effectively balancing local and global feature extraction (Liu et al., 2021). Thanks to these advantages, numerous studies have successfully applied it to medical imaging tasks. For example, Rana et al. used an improved Swin Transformer to classify chest X-ray images (Rana et al., 2025); by employing an enhanced autoencoder and the chaotic whale optimization algorithm for feature selection, the model achieved excellent performance in classifying chest diseases. Aldabayan et al. also applied a Vision Transformer-based diagnostic system to the multi-label detection of pneumonia and pneumothorax (Aldabayan, 2026). Their study systematically evaluated the diagnostic capabilities of ViT on the NIH ChestX-ray14 dataset and enhanced the interpretability of diagnoses through the visualization of attention mechanisms.

Recent studies have shown that combining the local feature extraction capabilities of CNNs with the global information modeling capabilities of Transformers can significantly improve the accuracy and robustness of medical image classification. MedViT, proposed by Manzari et al., employs LTB and ECB blocks designed for global and local feature extraction, respectively, and achieved classification accuracies of 0.981 and 0.951 on the PathMNIST and ChestMNIST datasets, respectively (Manzari et al., 2023). To address the clinical deployment needs for pneumonia classification, Nejat et al. proposed two hybrid architectures, MSAF-Net and DAR-Net, which achieved a balanced accuracy of 81.75% and an AUC of 0.9521, respectively, on a four-class pneumonia dataset (Nejat et al., 2026). Among these, MSAF-Net reduces model complexity by approximately 3.5 times, making it more suitable for resource-constrained clinical environments. Although the hybrid models mentioned above have achieved significant progress, how to continuously improve classification accuracy while further reducing hardware resource requirements remains an important area for optimization in this field.

Although the aforementioned hybrid architectures have demonstrated the advantages of integrating local and global features, most methods still face bottlenecks such as high model complexity and difficulty in deployment on resource-constrained devices, and they do not sufficiently account for the interaction between multiscale features. To address this issue, inspired by the hierarchical fusion approach of HiFuse (Huo et al., 2024), this paper proposes a novel lightweight medical image classification network, FLIFuse. Centered on a three-branch parallel hierarchical fusion architecture, this network aimed to achieve efficient integration of local details, global semantics, and multiscale context at a low computational cost. Specifically, the local feature extraction branch employed a lightweight ExtraDW (Qin et al., 2025) module designed to capture fine-grained local information such as lesion edges and textures, while the global feature extraction branch adopted a more lightweight Conv2Next module—inspired by the ConvNeXt (Liu et al., 2022) module—to model high-level semantics such as overall anatomical structures and image distributions. Building on this, this paper designed the FLI Block to adaptively fuse and facilitate cross-scale interaction between the multi-scale features output by the two branches, enabling the network to simultaneously perceive subtle changes in lesions and global contextual relationships, thereby improving classification accuracy while significantly reducing computational overhead.

Main Contributions of This Study:

1. Addressing the clinical need for precise identification of pneumonia pathogens, we innovatively designed a deep learning-based three-branch architecture to achieve accurate pneumonia classification. By simultaneously extracting global semantic information and local detailed features and efficiently fusing multi-scale features, the architecture more effectively utilizes imaging features at different granuralities, providing a new model framework for computer-aid interpretation of pneumonia imaging.

2. Lightweight Feature Extraction Architecture: The Conv2Next and ExtraDW modules were introduced to efficiently extract global semantic information and local detailed features, thereby reducing the number of model parameters and the number of floating-point operations (FLOPs), and balancing performance with computational resource consumption. This architecture helped the model maintain high classification capability while effectively controlling model size and computational complexity duringpneumonia classification, providing a more efficient model choice for deployment in resource-constrained environments.

3. Optimized Feature Fusion Module: The FLI Block was designed to adaptively fuse semantic information from features of different scales derived from the global and local feature extraction branches. This effectively addressed the issues of information complementarity and redundancy among multi-source, heterogeneous features. In classification tasks, this module helped the model more accurately identify and localize lesions, significantly enhancing its ability to distinguish between different types of pneumonia.

## 2 Methods

### 2.1 Overall architecture

The FLIFuse model, a lightweight network for classifying bacterial and viral pneumonia, employed a three-branch parallel architecture designed to efficiently extract and fuse local details and global semantics from X-ray images at a low computational cost, providing support for computer-aided interpretation of pneumonia imaging in resource-constrained settings. Its overall structure is shown in **Figure 1**. After preprocessing, the input image was processed by the lightweight global and local branches to extract features, respectively. The global branch captured macroscopic information such as lung field texture and lesion distribution, which helped distinguish between diffuse interstitial changes and segmental consolidation. The input image resolution was unified to 224x224, and both the global and loacal branches consisted of four stages, with the two branches maintaining the same spatial dimentions and number of channels at corresponding stages to facilitates subsequent fusion. The local branch focused on texture details and edge morphology, enhancing the response to subtle infiltrates or focal consolidation. In the FLI Block, global features used channel attention to dynamically calibrate channel weights, thereby enhancing sensitivity to key diagnostic features, while local features used spatial attention to highlight lesion areas and suppress background noise, facilitating the detection of minute lesions that are easily missed. The attention-processed features were integrated with the output from the FLI Block in the previous layer through a complementary balancing mechanism, which mitigated feature redundancy and complementary imbalances, thereby more comprehensively preserving the discriminative information regarding the differential expression between the two types of pneumonia. After concatenation, the features from the three branches were fed into a convolutional residual block for optimization, and the final result was output by the classifier.

**Figure 1:**
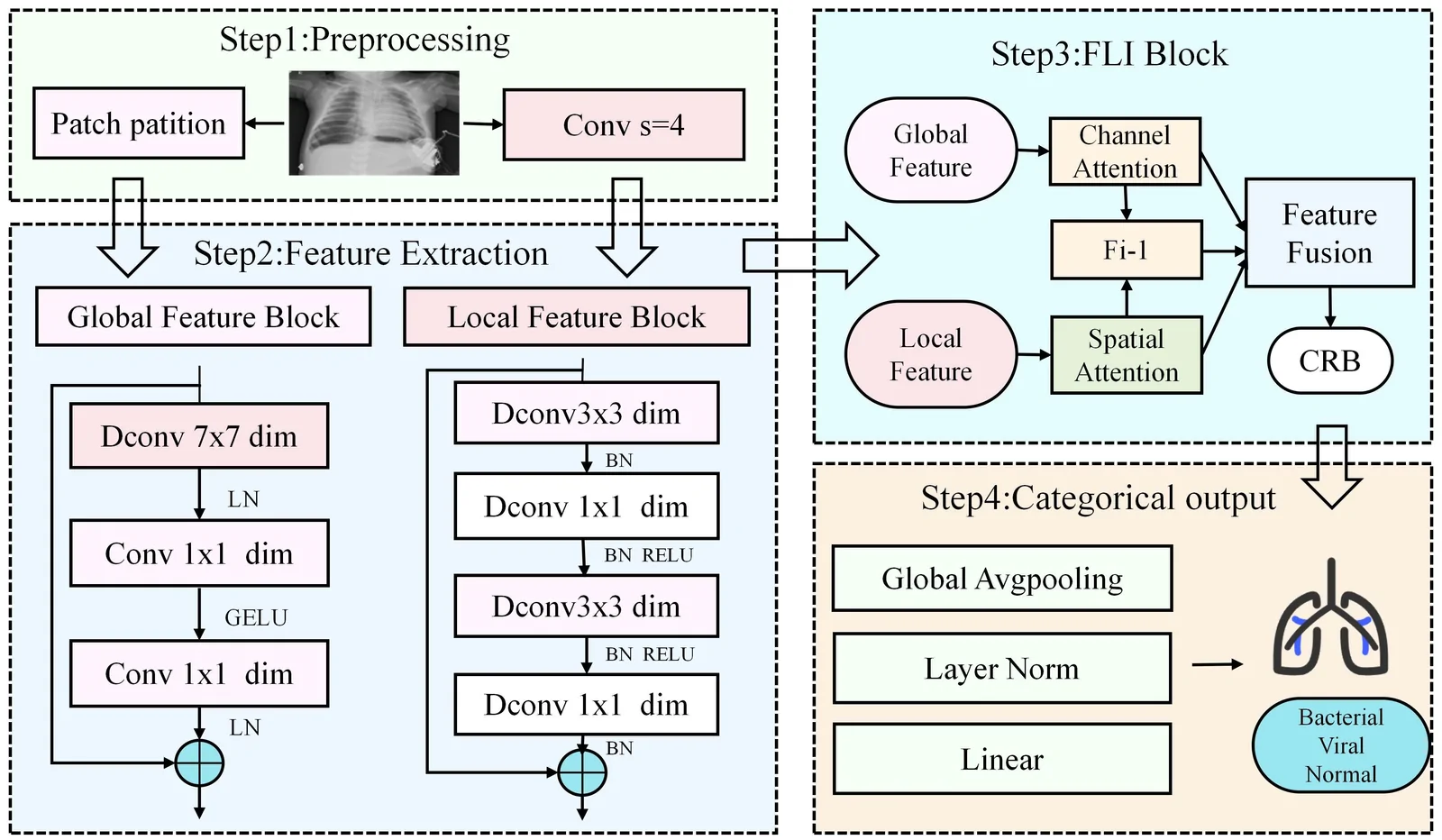
Overall structure of the FLIFuse model.

### 2.2 Global feature extraction branch

In the FLIFuse model, the global feature extraction branch was used to capture the global semantics and long-range dependencies in chest X-rays, helping the model obtain macroscopic information such as lung field texture and the overall distribution of lesions. This was valuable for distinguishing between diffuse interstitial changes and segmental/lobular consolidation. This branch employed multi-level feature extraction and downsampling operations to guide the model to focus on overall structural information; its structure is shown in **Figure 2**.

**Figure 2:**
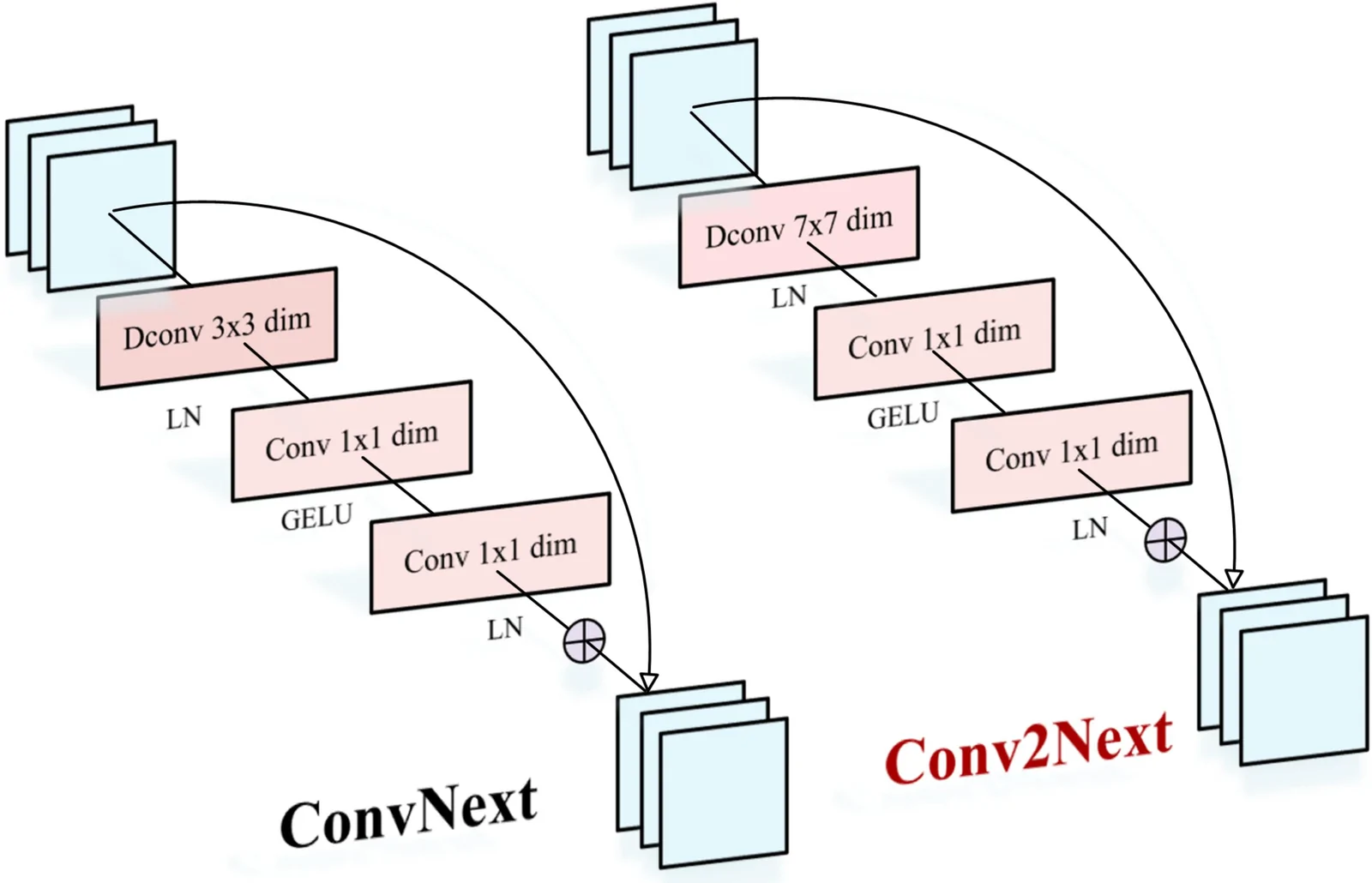
Global Feature Extraction Branch.

Specifically, when processing input medical images, the Patch Partition operation is performed first. This operation divides the image into multiple small patches, making it suitable for subsequent global feature extraction modules.The Patch Partition operation uses a 4x4 convolution with a stride of 4, mapping the input image into a 96-dimentional feature vector and producing an output map of size 56x56. Subsequently, the model employs Global Feature Blocks at different levels for feature extraction. The design of this Global Feature Block draws inspiration from the ConvNext model. However, to further reduce computational overhead, this study modifies ConvNext and introduces Conv2Next, achieving global feature extraction at minimal cost. The Conv2Next module consists of a large-kernel depthwise convolution, a 1x1 convolution, and a layer normalization in sequence, with a residual connection. The lightweight bottleneck in ConvNext lies in the fourfold channel expansion of the 1×1 convolution. Conv2Next compreses the channel scale to the point of no expansion, reducing redundant computation and parameters. Simultaneously, it replaces Dconv3×3 with deep convolutions featuring large kernels. Leveraging the low-channel nature of deep convolutions, the computational increase from enlarged kernel area is minimal. Yet, this approach directly expands the receptive field, enabling global feature capture in a single step. This design allows Conv2Next to inherit ConvNext’s strengths while minimizing computational cost to the extreme, achieving lightweight yet effective global feature extraction. Following each Global Feature Block, a stride-2 convolution and layer normalization operation are applied. The stride-2 convolution serves as a downsampling mechanism, reducing data dimensions while facilitating the extraction of deeper features. Layer normalization standardizes data distribution, further enhancing the model’s ability to express global features.

### 2.3 Local feature extraction branch

The local feature extraction branch was used to capture fine-grained texture and edge information in chest X-rays, which helped enhance the model’s sensitivity to lesion details while maintaining computational efficiency. This study adopted the ExtraDW module from MobileNetV4 to enhance local feature extraction capabilities, as shown in **Figure 3**. The ExtraDW module consisted of a 3x3 depthwise convolution, a 1x1 convolution, batch normalization, and a ReLU activation function in sequence, where the 3x3 depthwise convolution was used to extract spatial detail features, the Ixl convolution was used for cross-channel information fusion, batch normalization was used to stabilize the training process, and the ReLU activation function introduced nonlinearity. In the task of classifying chest X-ray images of pneumonia, the ExtraDW module demonstrated significant advantages in extracting detailed features due to its depthwise separable convolution structure. By decomposing traditional convolutional operations into depthwise convolutions and point-wise convolutions, this module significantly reduced computational complexity while enabling more precise capture of subtle lesion changes, such as ground-glass opacities and small nodules—early signs of pneumonia. Point-wise convolution effectively enhanced the fusion of cross-channel features, suppressed noise interference, and improved the robustness of feature representations. By introducing the ExtraDW module, this study enhanced the model’s sensitivity to subtle abnormalities and classification accuracy, while optimizing performance and maintaining high efficiency. Finally, the extracted local features were fed into the FLI Block and fused with the global information extracted by the Globe Feature Path, further enhancing the model’s discriminative capability. This design ensured that the model could accurately identify lesion areas in medical image classification tasks and, by complementing global information, improved classification accuracy and robustness.

**Figure 3:**
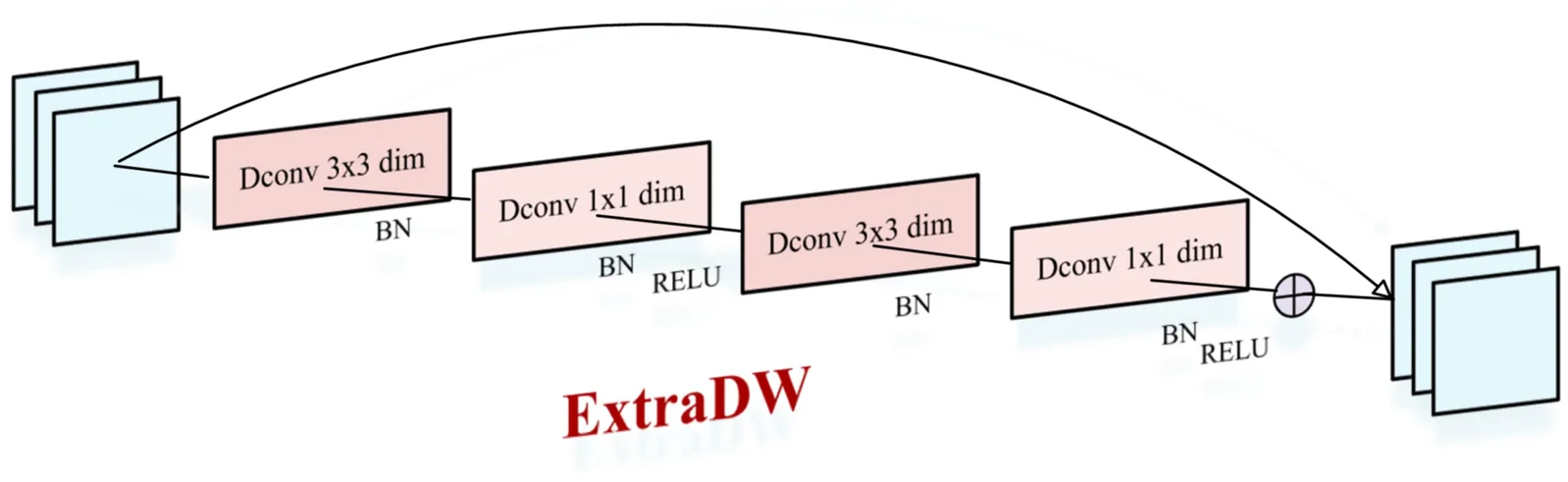
ExtraDW Feature Extraction Branch.

### 2.4 FLI block

The adaptive feature fusion module employed a three-stage collaborative fusion mechanism. First, global features obtained through the global feature extraction branch were fed into the channel attention module. This module dynamically calibrated the response weights of each channel by modeling inter-channel dependencies, enabling the model to adaptively emphasize global feature channels more valuable for classification discrimination. This helped capture differences between bacterial and viral pneumonia at the macro level, such as the distribution of lesions and lung field texture. Second, local features were fed into a spatial attention module to precisely highlight lesion-related detail regions in the image while suppressing background noise, thereby enhancing the model’s response to subtle imaging manifestations such as ground-glass opacities and small nodules. Finally, through a feature complementarity and balancing mechanism, the features processed by the two attention mechanisms described above were integrated at multiple scales with the output from the adaptive feature fusion module in the previous layer. As shown in **Figure 4**, this fusion process not only achieved synergistic optimization across global semantic modeling, detail enhancement, and information integration but also effectively mitigated issues such as gradient vanishing, gradient explosion, and network degradation by incorporating the CRB module, thereby significantly enhancing the network’s ability to capture global and local information at all levels.

**Figure 4:**
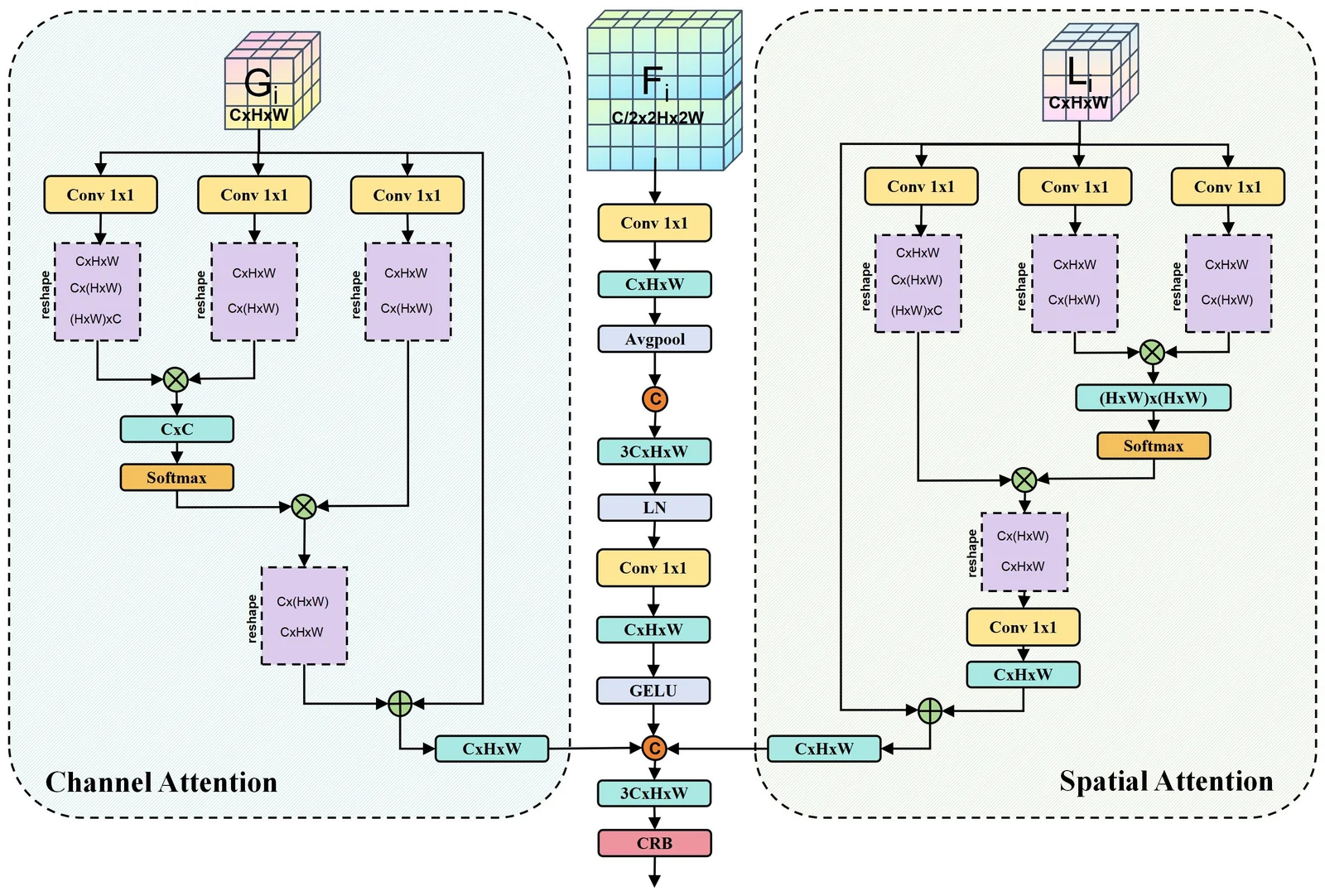
FLI Block Structure.

The channel attention mechanism aims to significantly enhance the response capability to semantically relevant features by modeling interdependencies between channels. This approach amplifies the weights of important channels while suppressing redundant information. Specifically, using the global feature Gi as input, channel-level features are first efficiently extracted through multiple pointwise convolutions, where three 1x1 concolutions generate query, key, and value features, respectively. Subsequently, reshaping operations transform these features into a matrix structure suitable for channel attention computations. Building upon this, the softmax function generates attention weights along the channel dimension, precisely highlighting key channels while suppressing redundant information. Finally, feature reconstruction restores the original form, simultaneously strengthening the model’s ability to capture global semantic structures and achieving dynamic enhancement of important channel features alongside effective filtering of redundant information. This significantly improves the specificity and discriminative power of feature representation. As shown in Equations 1, 2, *i* denotes the index of the query position, *j* enumerates all possible positions, $f\left(x_{i},x_{j}\right)$ represents the relationship between positions *i* and *j*, and *C*(*x*) is a normalization factor. **W***_q_*, **W***_k_*, **W***_w_* and **W***_z_* all denote 1×1 convolutional measurements. For simplicity, all reshape operations are omitted in the equations; the same applies to the spatial attention calculation formulas below.

(1) $$\frac{f\left(x_{i},x_{j}\right)}{C\left(x\right)}=\frac{W_{q}x_{i},\;W_{k}x_{j}}{N_{p}}=\frac{{\left\{W_{q}x_{i}\right\}}^{⊤}\cdot W_{k}x_{j}}{N_{p}}$$

(2) $$y_{i}=x_{i}+W_{z}\sum_{j=1}^{N_{p}}\frac{f\left(x_{i},x_{j}\right)}{C\left(x\right)}\left(W_{v}\cdot x_{j}\right)$$

The Spatial Attention Module is designed to enhance the expressive power of critical spatial positions within feature maps extracted from the Local Feature Extraction Module. This module aims to significantly strengthen the representation of key spatial information in feature maps, thereby more effectively capturing local detail features in images while suppressing interference from irrelevant regions. Taking the output of the local feature extraction branch as input, it first performs feature transformations through multiple 1×1 convolutions and reshapes the results into a form suitable for matrix operations. Subsequently, matrix multiplication constructs an HW×HW spatial correlation matrix to precisely capture complex dependencies between different spatial locations. After softmax normalization, this spatial attention map is used to weight and reconstruct the input features, thereby highlighting salient regions and effectively suppressing redundant information. The final enhanced feature map not only preserves the original spatial resolution but also gains enhanced spatial perception capabilities. This provides stronger support for subsequent feature fusion and recognition tasks, significantly improving task accuracy.As shown in Equations 3, 4, *i* denotes the index of the query position, *j* enumerates all possible positions, $f\left(x_{i},x_{j}\right)$ represents the relationship between positions *i* and *j*, and *C*(*x*) is a normalization factor. **W***_q_*, **W***_k_* and **W***_v_* all denote 1×1 convolutional operations.

(3) $$\frac{f\left(x_{i},x_{j}\right)}{C\left(x\right)}=\frac{W_{q}x_{i},\;W_{k}x_{j}}{N_{p}}=\frac{W_{q}x_{i}\cdot {\left\{W_{k}x_{j}\right\}}^{⊤}}{N_{p}}$$

(4) $$y_{i}=x_{i}+\sum_{j=1}^{N_{p}}\frac{f\left(x_{i},x_{j}\right)}{C\left(x\right)}\;\left(W_{v}\cdot x_{j}\right)$$

Finally, the fusion features ***F****_i_*_−1_ from the preceding FLI module undergo pointwise convolution and average pooling to align dimensions and compress information, ensuring perfect adaptation to the current layer’s fusion scale. These features are then concatenated with Gi and Li, and stabilized through Layer Normalization to ensure model training stability. Next, GELU and 1×1 convolutions further enhance semantic expression capabilities, finely tuning the importance of feature channels to select more discriminative representations. Finally, features from the three branches are concatenated, and the resulting feature input is fed into the CRB for final optimization.

## 3 Experiments

### 3.1 Dataset

ChestXRay2017 (Kermany, 2018) is an open-source medical dataset released by the Guangzhou Maternal and Child Health Center. It contains 5,856 chest X-ray images of pediatric patients aged 1 to 5 years, covering three categories: normal, bacterial pneumonia, and viral pneumonia. All images underwent rigorous screening and were graded by professional physicians to ensure quality. Primarily designed to train deep learning models for assisting physicians in pneumonia detection, this dataset enhances diagnostic efficiency and accuracy. It is available for download via the Mendeley Data platform. By providing high-quality data support for medical image analysis, it facilitates the development of efficient diagnostic assistance tools.

COVID-19 CHEST X-RAY DATABASE (Chowdhury et al., 2020) is a chest X-ray image dataset designed for classification tasks, specifically to distinguish COVID-19 positive cases, normal cases, and viral pneumonia cases. The dataset includes 1,200 COVID-19 positive images, 1,341 normal images, and 1,345 viral pneumonia images. COVID-19 images were collected from multiple public sources such as GitHub, German medical schools, and SIRM, while normal and viral pneumonia images originate from Kaggle’s Chest X-Ray Images (pneumonia) dataset. All images are provided in PNG format with resolutions of either 1024×1024 pixels or 256×256 pixels. This dataset serves as a valuable reference resource for researchers engaged in COVID-19 classification studies.

Nerthus (Pogorelov et al., 2017) is an endoscopic bowel preparation assessment dataset specifically designed to enhance colonoscopy efficiency. It comprises 21 videos totaling 5,525 frames, annotated and validated by experienced endoscopists, and graded on a 0–3 scale according to the Boston Bowel Preparation Scale. The dataset is freely available for download via its official website, aiming to advance machine learning algorithm development. It assists clinicians in achieving objective, automated assessment of bowel preparation, thereby improving diagnostic efficiency and accuracy while providing standardized data support for medical imaging research.

### 3.2 Evaluation metrics

This study focused primarily on accuracy and evaluated the model by considering the number of parameters and floating-point operations (FLOPs) to assess the model’s lightweight degree in terms of computational resources, providing a basis for future deployment research in resource-constrained environments. Additionally, the study considered three metrics: precision, recall, and F1 score. In the fields of scientific research and data analysis, we typically use the following terms to describe the performance of classification models: True Positive (TP) refers to samples that are actually affected by the condition and are correctly predicted. A True Negative (TN) is a sample that is not actually in the target class but is correctly predicted as such. A False Positive (FP) refers to a sample that is not actually in the target class but is incorrectly predicted as belonging to the target class. A False Negative (FN) refers to a sample that is actually affected by the condition but is missed by the model.

ACC stands for Accuracy, a metric used to evaluate the performance of classification models. Accuracy refers to the proportion of samples correctly predicted by the model out of the total number of samples, and it provides an intuitive measure of the model’s overall ability to distinguish between bacterial and viral pneumonia. Accuracy can be calculated using the Equation 5:

(5) $$\text{ACC}=\frac{\text{TP}+\text{TN}}{\text{TP}+\text{TN}+\text{FP}+\text{FN}}$$

Precision measures the proportion of samples that the model predicts to belong to a certain category and that actually do belong to that category. In the context of pneumonia classification, high precision means fewer cases are misclassified as bacterial pneumonia, which helps reduce unnecessary antibiotic use. The formula is shown in Equation 6:

(6) $$\text{Precision}=\frac{\text{TP}}{\text{TP}+\text{FP}}$$

Recallis equivalent to sensitivity, which measures the proportion of samples that actually belong to a certain category and are correctly identified by the model. A high recall rate indicates a lower risk of missed diagnoses and is of significant clinical value in ensuring timely detection of patients with bacterial pneumonia and avoidance of treatment delays. Specifically measures the proportions of normal background pixels that are correctly identified, reflecting the model’s ability to suppresses false positivities.A high specifically indicates that the model can effectively distinguish normal tissue from leision regions. The formulas are given in Equations 7, 8:

(7) $$\text{Recall}=\frac{\text{TP}}{\text{TP}+\text{FN}}$$

(8) $$\text{Specificity}=\frac{\text{TN}}{\text{TN}+\text{FP}}$$

The F1 score is the harmonic mean of precision and recall, striking a balance between the two. When a dataset suffers from class imbalance, the F1 score provides a more robust reflection of model performance than precision. Therefore, this study incorporated it into the evaluation framework to assist in assessing the model’s overall discriminative ability. Its formula is given in Equation 9:

(9) $$F_{1}=\frac{2\text{ TP}}{2\text{ TP}+\text{FP}+\text{FN}}$$

The statistics of the number of parameters and computational cost were based on the PyTorch framework and the thop library, with the input image resolution fixed at 224×224. For each layer in the model, the number of learnable parameters Param*_i_* and the number of floating-point operations FLOPs*_i_* were counted, and then summed over all layers with unit conversion. The number of parameters is expressed in millions and is used to estimate the storage overhead of the model. FLOPs are expressed in billions and represent the number of floating-point operations required for one forward inference, which is directly related to inference latency. The calculations for convolutional and fully connected layers follow standard rules. The calculation formulas for both are given in Equations 10, 11:

(10) $$\text{Parameters}\left(\text{M}\right)=\frac{1}{10^{6}}\sum_{l=1}^{L}{\text{Param}}_{l}$$

(11) $$\text{FLOPs}\left(\text{G}\right)=\frac{1}{10^{9}}\sum_{l=1}^{L}{\text{FLOPs}}_{l}$$

### 3.3 Experiments setting

All images from the public dataset used in this study were included in the experiments. Each dataset was split into 70% training set and 30% test set, and 20% of the training set was further divided as a validation set for simultaneous validation. All splits were performed at the patient level to effectively avoid data leakage. All images were uniformly resized to 224×224 pixels using bilinear interpolation, and pixel values were normalized to [-1, 1]. The experiments were conducted on a computer running Windows 10 Pro (64-bit), configured with a 12th Gen Intel^®^ Core™ i5-12400F 2.50 GHz CPU, Kingbank DDR4 3200MHz 16GBx2memory, and an NVIDIA GeForce RTX 4070 GPU.

The software environment included Python 3.7.12, PyTorch 1.12.Le and CUDA 11.6. During training. random horizontal flipping was applied for data augmentation, and all augmentation operations were applied only to the training set. to 8, the total number of epochs to SGD 1000, and stochastic gradient descent (SD) was used as the optimizer with a random seed of 42, an initial learning rate of 0.001momentum of 0.9, and weight decay of 0.01. A cosine annealing schedule was adopted for the learning rate, and the loss function was cross entropy loss.

### 3.4 Results on ChestXRay2017

In this study, to comprehensively evaluate the performance of the FLIFuse model on the pneumonia classification task, we compared it with several state-of-the-art architectures, including MobileNetV4, EfficientNet (Tan and Le, 2020), EfficientNetV2 (Tan and Le, 2021), ConvNext, HiFuse, ViT, MobileViT (Mehta and Rastegari, 2022), Swin Transformer, and FasterNet. All experiments were conducted under the same conditions, and the:results are shown in **Table 1**. The FLIFuse model demonstrated significant technical advantages in the comprehensive performance evaluation. In terms of model lightweight characteristics, this architecture employed an innovative design that achieved a balance between computational efficiency and accuracy. Its computational complexity was only 6.612 GFLOPs and the parameter size was controlled at 33.428 M, which substantially reduced resource consumption while maintaining high performance. This, gives it considerable deployment potential in computationally resource-constrained scenario and this metric was notably superior to that of mainstream architectures such as EfficientNetV2, ConvNext HiFuse, and Swin Transformer. In terms of recognition performance, FLIFuse achieved an accuracy of 91.4%, representing a significant improvement over similar lightweight models such as MobileNetV4 and EfficientNet, and providing a relatively reliable auxiliary judgement method for rapidly distinguishing normal, bacterial, and viral pneumonia, thereby laying a foundation for subsequent clinical validation.

**Table 1:** Performance comparison of the ChestXRay2017 dataset.

Model	GFLOPs	Param (M)	Acc (%)	Re (%)	Pre (%)	F1 (%)
MobileNetV4	2.170	31.160	90.2	70.1	74.8	72.0
Efficientnet	5.328	63.797	89.4	69.0	74.1	71.0
EfficientNetV2	12.367	117.239	90.0	69.3	74.1	71.3
ConvNext	15.354	87.514	91.0	70.5	74.8	72.3
HiFuse	20.239	132.311	91.4	71.4	75.7	73.2
ViT	16.863	85.648	89.9	69.9	74.8	71.9
MobileViT	1.464	4.940	88.7	67.3	73.0	69.6
Swin Transformer	15.169	86.684	91.0	71.2	75.5	73.0
FasterNet	1.916	13.704	89.5	68.4	73.4	70.5
FLIFuse	6.612	33.428	91.4	72.7	76.8	74.4

To evaluate the applicability of the model in resource-constrained environments, we supplemented two additional metrics: weight file size and peak activation memory. The model weight file size is calculated by multiplying the total number of parameters by the wight storage precision. Under INT8 quantizization, the wight file size of our model is approximately 33.4 MB, which is about 74.7% lower that the 132.3 MB of the baseline model. The peak activation memory is estimated based on the output feature map sizex of each layer and the main intermediate tensors involved in the attention computation. Since the input resolution is 224x224, the batch size during actual interface is typically 1, and the precision is FP32, the peak activation memory of our model is estimated to be approximately 5–10 MB, which is at a relatively low level and reflects the small memory overhead during model runtime. The above results demonstrate the lightweight advantages of the model in terms of both storage and runtime memory.

Additionally, to more intuitively demonstrate the comprehensive advantages of the FLIFuse model in terms of accuracy, parameter count, and computational complexity, this study plotted corresponding scatter plots using Acc, Param, and GFLOPs as metrics, as shown in **Figure 5**. This set of charts systematically presents the outstanding performance of the FLIFuse model in pneumonia classification tasks by examining the multidimensional relationship among computational load, parameter scale, and classification performance. **Figure 5a** focuses on the correlation between accuracy and computational load, clearly showing that FLIFuse maintains a high accuracy of 91.4% while requiring significantly lower computational costs than high-performance models like HiFuse. This low computational load characteristic enables fast interference without relying on high-end hardware, making it suitable for environments with limited computing power. **Figure 5b** focuses on the balance between accuracy and parameter scale. FLIFuse achieves superior classification accuracy with significantly fewer parameters than classic models such as ConvNext and Swin Transformer, which means that it can effectively learn discriminative related to pneumonia while saving storage resources, reflecting a favorable balance between parameter scale and discriminative capability.

**Figure 5:**
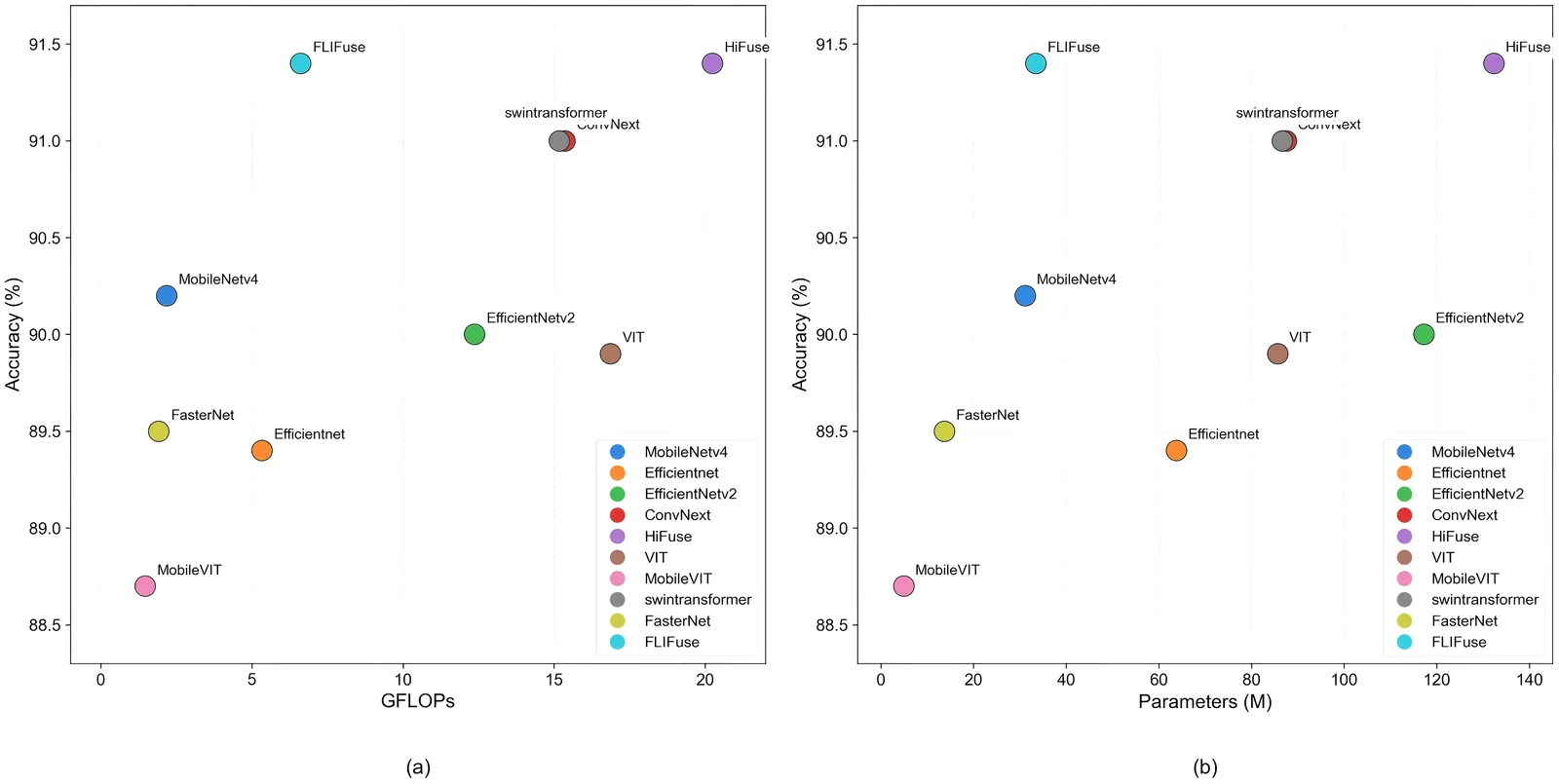
Accuracy comparison with respect to GFLOPs and parameters. **(a)** Accuracy vs. GFLOPs; **(b)** Accuracy vs. Parameters.

To further examine the regions the model focuses on and the basis for its decisions during classification, we used the Grad-CAM method to visualize and analyze the predictions of FLIFuse The Grad-CAM visualization was based on the trained FLIFuse model The target layer was selected as the depthwise convolutional layer of the last local block in the final stage. The test image was fed into the model, and backpropagation was performed for the predicted class to obtain the activation maps and gradients of the target layer. Global average pooling was applied to the gradients to obtain channel weights, which were then used to compute a weighted sum of the activation maps After applying ReLU and normalization, a heatmap was generated and up sampled to the original image size and overlaid on the original image. The visualization images were taken from the test set, and representative samples were selected for display. In the heatmaps, warm-colored regions represent high-response areas during model classification. If these regions are concentrated at the lesion locations in the lungs, it indicates that the model used effective pathological features for discrimination.

The Grad-CAM heatmap visualization results for the three classes of the ChestXRay2017 dataset are shown in **Figure 6**. In each column, the original chest X-ray image is displayed at the top, and the overlaid feature heatmap is shown below. **Figure 6a** corresponds to a normal chest X-ray sample, where no concentrated high-response regions are formed within the lung fields, which is consistent with the imaging characteristics of normal lungs, namely uniform lung markings and the absence of inflammatory infiltration. **Figures 6b** shows a correctly predicted viral pneumonia sample with a model confidence of 98.31%. The highlighted regions in the heatmap are mainly concentrated in areas of pulmonary interstitial exudation and diffuse ground-glass opacities, with a relatively diffuse activation range. **Figure 6c** shows a correctly predicted bacterial pneumonia sample with a confidence of 95.93%. The heatmap exhibits more concentrated focal high-response hotspots, which are largely consistent with the locations of lobar or segmental consolidation lesions. These results indicate that the feature regions attended by the model are in good agreement with the typical imaging manifestations of different pneumonia types, demonstrating the interpretability of the model in lesion localization and class discrimination.

**Figure 6:**
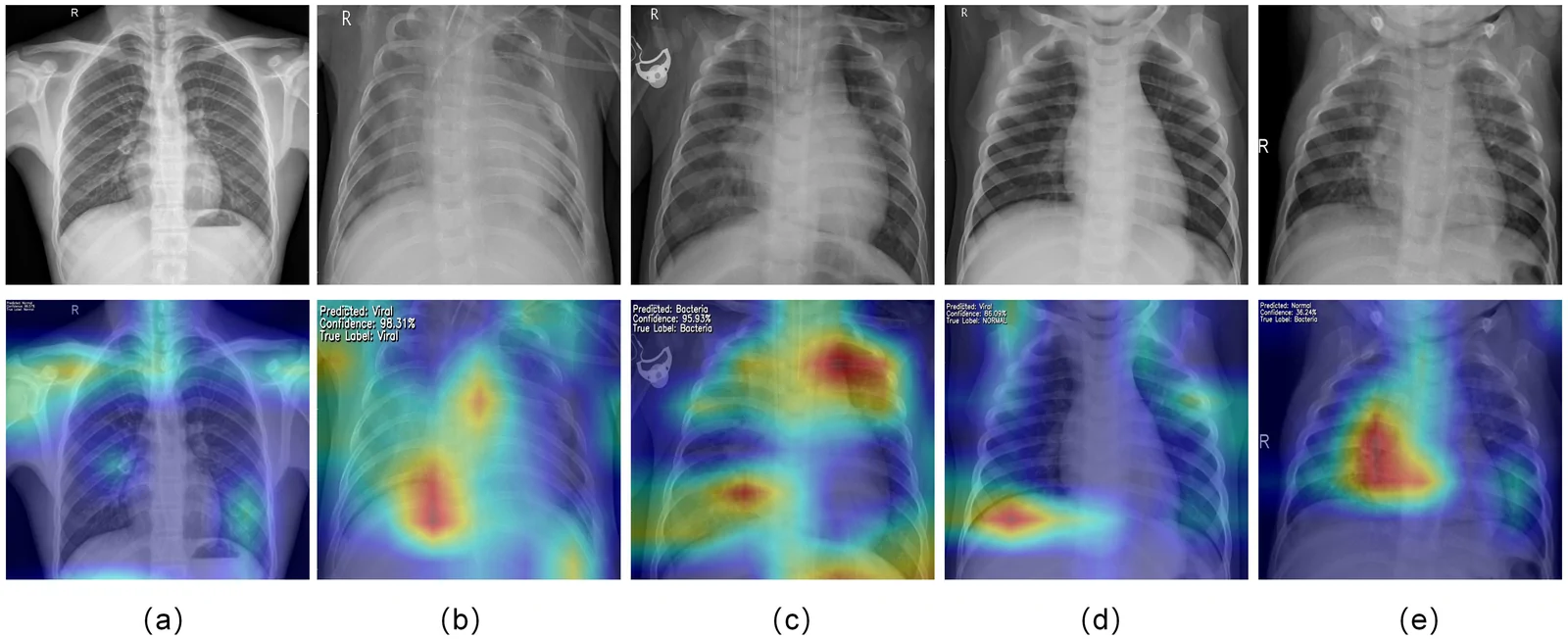
Grad-CAM visualization results for representative samples in the ChestXRay2017 dataset: **(a)** normal; **(b)** viral; **(c)** bacterial; **(d)** false positive; **(e)** false negative.

In misclassified samples, the heatmap also provide valuable analytical clues. **Figure 6d** shows a false positive case, where the model classified a normal chest X-ray as viral pneumonia. The heatmap shows that the model produced a relatively strong response to local normal lung markings, indicating that the model is still sensitive to certain texture patterns. Such misclassifications can be further reduced through training strategies in future work. **Figure 6e** be shows a false negative case, where the true label was bacterial pneumonia, but the heatmap shows visible activation in the lesion region, although the response intensity did not reach the classification threshold. This indicates that the model has a certain degree of lesion perception ability. In the future, threshold optimization or multi-scale features can be combined to further improve the recognition stability for low-confidence cases. Overall, the Grad-CAM visualization not only demonstrates the decision-making basis of the model but also provides intuitive support for its auxiliary reference value in clinical image reading.

### 3.5 Results on COVID-19 chest x-ray database

To further validate the effectiveness of FLIFuse in pneumonia-related classification tasks, this section presents experiments conducted on the representative COVID-19 chest X-ray database and compares it with benchmark models such as MobileNetV4 and FasterNet in terms of accuracy, recall, precision, and F1 score. The results are shown in **Table 2**. On this dataset, FLIFuse achieved an accuracy of 88.1%, a recall of 78.8%, a precision of 83.3%, and an F1 score of 80.4%, outperforming models such as,ConvNext, EfficientNet, ViT, HiFuse, and MobileNetV4 in overall performance. In particular, the 78.8% indicates that the model can identify the majority of COVID-19-positive cases, helping to reduce the risk of missed diagnoses during screening, while the precision of 83.3% indicates that the model has a relatively low flase positive rate for negative samples. These results demonstrate that FLIFuse possesses certain discriminative capability in distinguishing COVID-19-positive, normal, and viral pneumonia images, and can serve as a reference for X-ray-based auxiliary screening of pneumonia.

**Table 2:** Performance comparison of the COVID-19 chest x-ray database.

Model	Acc (%)	Re (%)	Pre (%)	F1 (%)
MobileNetV4	86.9	75.6	81.6	77.6
Efficientnet	85.3	73.5	79.6	75.6
EfficientNetV2	87.4	75.9	81.8	78.0
ConvNext	87.9	77.7	82.9	79.5
HiFuse	87.6	76.8	82.0	78.8
ViT	87.1	75.3	80.6	77.3
MobileViT	84.3	71.1	78.4	73.6
Swin Transformer	87.4	75.5	81.4	77.6
FasterNet	86.6	75.1	81.4	77.4
FLIFuse	88.1	78.8	83.3	80.4

As shown in **Figure 7**, FLIFuse achieves the highest levels across the four core metrics of accuracy, recall, precision, and F1 score, with relatively consistent bar heights, making it a typical well-balanced model obvious weaknesses. In contrast, while ConvNext is somewhat competitive on certain metrics, it lacks consistency and models such as HiFuse exhibit noticeable function. This multidimensional balance indicates that FLIFuse archives good overall recognition performance in pneumonia classification task and performs particularly well on the F1 score, which represents comprehensive performance, thereby providing a foundation for subsequent clinical validation.

**Figure 7:**
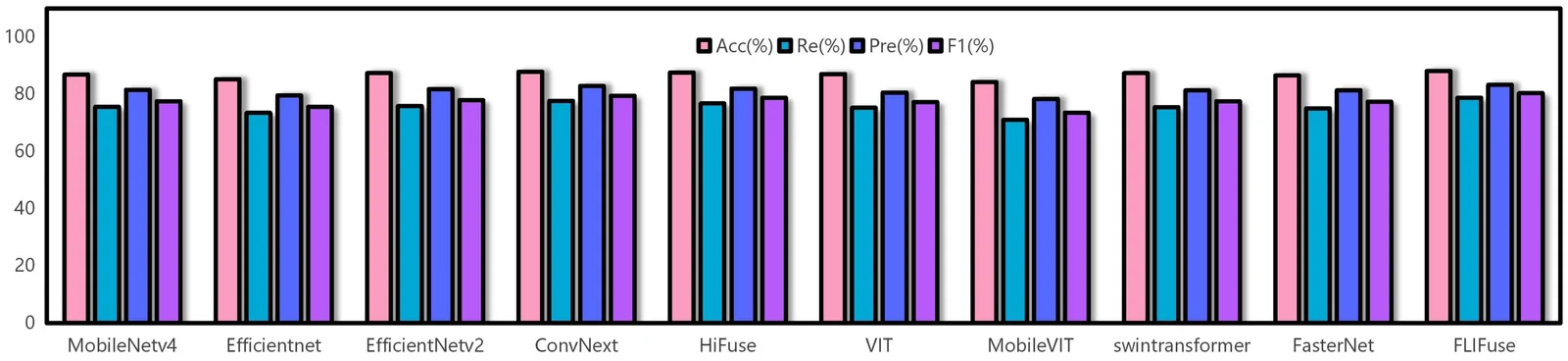
Model performance comparison on COVID-19 chest X-ray dataset.

**Figure 8** presents the visualization results of several representative samples from the COVID-19 Chest X-ray Database, which are used to observe the attention patterns of the model on this dataset. **Figure 8a** corresponds to a correctly predicted normal chest X-ray with a model confidence of 99.81%.The heatmap does not show high-intensity concentrated activation within the lung fields, with only weak responses at the chest wall margins and mediastinal region, which is consistent with the imaging appearance of normal lungs. **Figure 8b** shows a correctly predicted case of ordinary viral pneumonia with a confidence of 99.70%. The highlighted regions in the heatmap are diffusely distributed along the middle and lower lung fields, with relatively blurred boundaries, which is consistent with the imaging characteristics of viral pneumonia involving the pulmonary interstitium. **Figure 8c** shows a correctly predicted COVID-19 pneumonia sample with a confidence of 91.47%. The heatmap exhibits a wider range of multifocal patchy activation, predominantly distributed in the peripheral zones. This activation pattern shares similarities with that of ordinary viral pneumonia, while alse showing certain differences in distribution.

**Figure 8:**
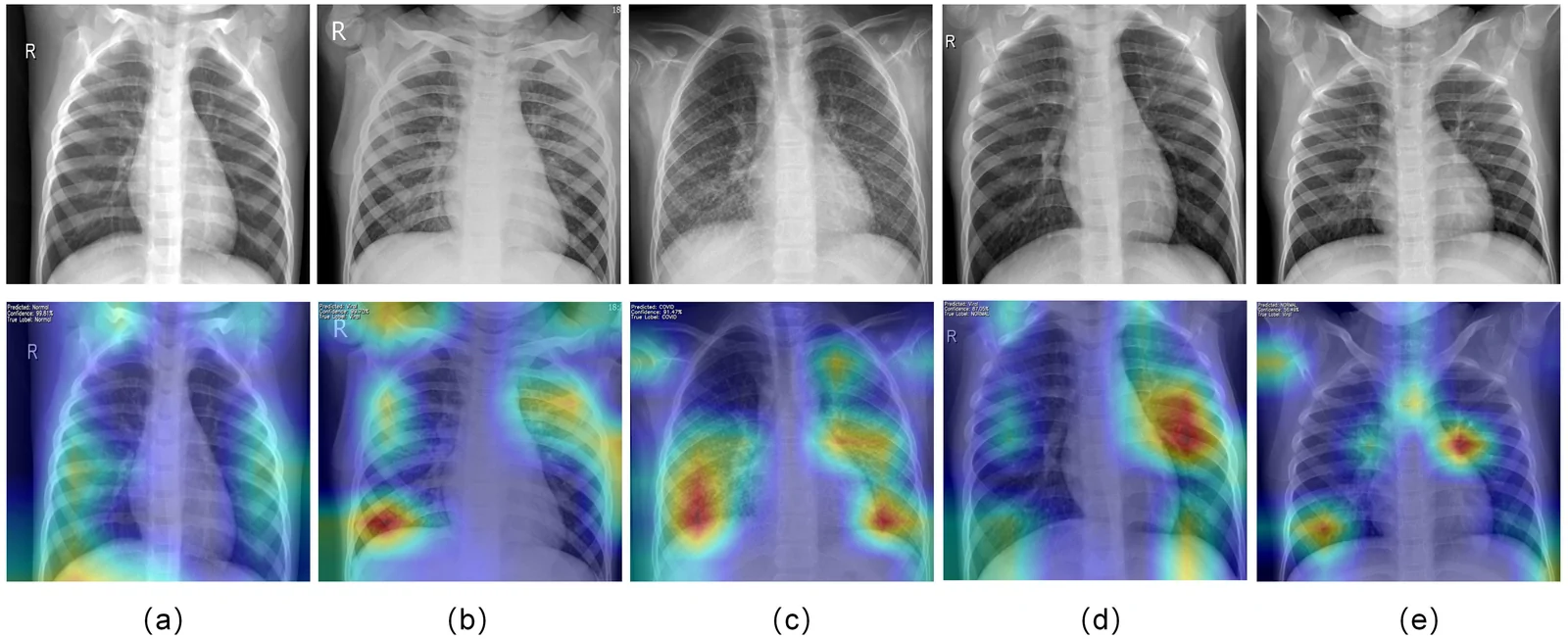
Grad-CAM visualization results for representative samples in the COVID-19 CHEST X-RAY DATABASE dataset: **(a)** normal; **(b)** viral; **(c)** COVID-19; **(d)** false positive; **(e)** false negative.

In the misclassified samples. **Figure 8d** shows a false positive case in which a normal chest X-ray was misclassified as viral pneumonia. The heatmap shows that the model produced a relatively strong response to local normal lung markings, indicating that the model is overly sensitive to such texture patterns. In future work, targeted constraints can be introduced during training to alleviate this type of bias. **Figure 8e** shows a false negative case where the true label was viral pneumonia, but the model output the normal class. However, the heatmap shows visible activation signals in the lesion region, indicating that the model has preliminarily captured lesion-related cues, but the decision threshold was relatively high. In future work, the utilization of weak features can be enhanced by adjusting the classification boundary or introducing multi-scale context. Overall, the heatmaps on this dataset can reflect the model’s attention patterns to pulmonary inflammatory lesions and the differences in recognition under varying degrees of lesion severity, providing a certain degree of interpretability reference for assisting clinical differentiation of pneumonia types.

### 3.6 Results on nerthus

To evaluate the architectural transferability of the FLIFuse model across different medical imaging modalities, this study introduced the Nerthus dataset as a cross-domain test set for systematic evaluation. For model comparison, state-of-the-art benchmark models such as MobileNetV4 and FasterNet were selected and comparisons were conducted across multiple dimensions including accuracy, recall, precision, and F1 score, as shown in **Table 3**. The Nerthus dataset contains real clinical endoscopic images and covers various bowel preparation grades, providing a reliable basis for examining the adaptability of the model’s feature extraction capability across different medical image domains. Comparison with multiple state-of-the-art models helps highlight performance advantages and characteristics of the FLIFuse model. Combining the number of parameters and GFLOPs in **Table 1** with the evaluation results in **Figure 5** the good architectural transferability and high efficiency of the FLIFuse model in cross-domain medical imaging tasks can be clearly demonstrated.

**Table 3:** Performance comparison of the nerthus dataset.

Model	Acc (%)	Re (%)	Pre (%)	F1 (%)
MobileNetV4	93.1	80.0	83.0	81.3
Efficientnet	93.5	79.4	82.2	80.7
EfficientNetV2	93.8	80.9	83.7	82.1
ConvNext	94.0	81.7	84.2	82.7
HiFuse	94.6	82.7	85.2	83.8
ViT	94.9	83.4	85.6	84.4
MobileVit	91.1	77.0	81.1	78.7
Swin Transformer	94.4	81.0	83.5	82.1
FasterNet	92.4	78.4	81.8	79.7
FLIFuse	94.7	84.8	87.1	85.8

As shown in **Figure 9**, the FLIFuse bars are largely at the highest positions. Compared with lightweight models such as MobileNetV4, EfficientNet, MobileViT, and FasterNet, FLIFuse achieved significant improvements in key metrics including accuracy, recall, precision, and F1 score. When compared with high-precision models such as EfficientNetV2, ConvNext, HiFuse, ViT, and Swin Transformer, FLIFuse demonstrated clear advantages recall, precision, and F1 score while substantially reducing the number of parameters and computational complexity, with the exception that its accuracy 0.2% lower than that of ViT. It is worth noting that FLIFuse not only performed excellentlyin the pneumonia image classification task but also maintaned good classification performance in other medical image classification tasks, indicating that the model has good generalization ability across different medical image classification tasks.

**Figure 9:**
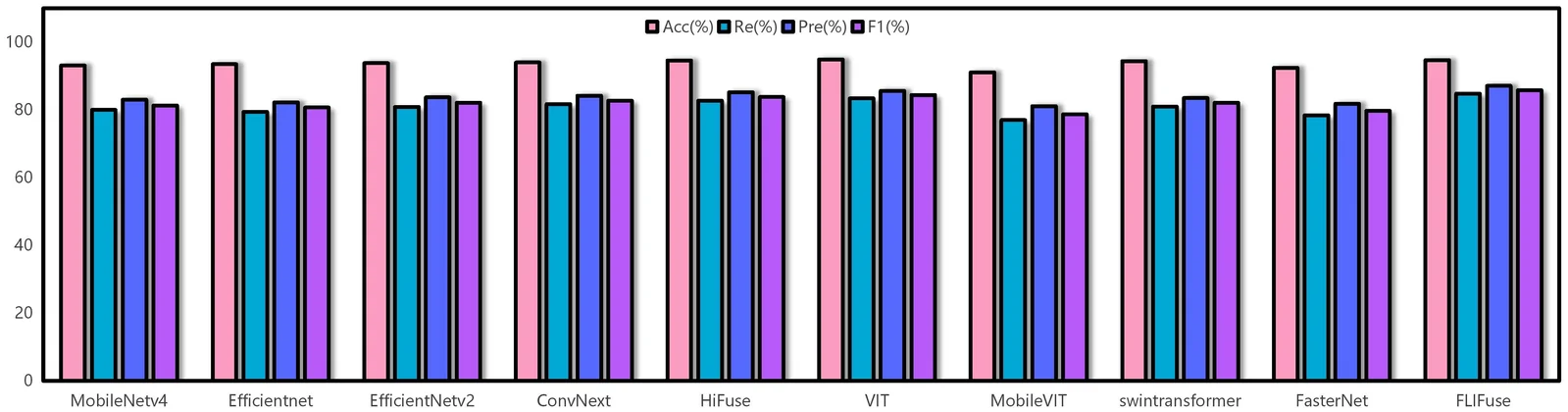
Model performance comparison on Nerthus dataset.

### 3.7 Statistical robustness analysis

To further examine the stability of the model performance, we conducted five-fold cross-validation for FLIFuse and HiFuse on the Chest XRay2017, COVID-19, and Nerthus datasets, and the results are shown in **Table 4**. Overall, the mean values of the main metrics for FLIFuse on the three datasets are all higher than those of HiFuse, and the standard deviations of most metrics are similar to or smaller than those of HiFuse, indicating relatively stable performance. On the ChestXRay2017 dataset, FLIFuse achieved an accuracy of 91.40%, an F1 score of 78.11%, a recall of 76.73%, and a specificity of 95.20%, with all mean values outperforming HiFuse. Although some confidence intervals overlap to a certain extent, the mean advantage remains consistent across different folds. On the COVID-19 dataset, FLIFuse achieved an accuracy of 88.02% and an F1 score of 80.20%, with recall and specificity also comprehensively leading over HiFuse. Although the confidence intervals show slight overlap, the overall trend remains unchanged. Since both datasets consist of chest X-ray images, the above results collectively indicate that FLIFuse has a relatively stable performance advantage in pneumonia-related classification tasks.

**Table 4:** Performance comparison between FLIFuse and HiFuse based on five-fold cross-validation.

Dataset		Acc (%)		Re/Sensitivity (%)		Pre (%)		F1 (%)		Specificity	
	Model	Mean ± SD	95% CI	Mean ± SD	95% CI	Mean ± SD	95% CI	Mean ± SD	95% CI	Mean ± SD	95% CI
ChestXRay2017	HiFuse	90.18 ± 1.13	[88.77,91.58]	74.60 ± 3.51	[70.24,78.96]	75.02 ± 0.69	[74.17,75.88]	74.45 ± 1.74	[72.29,76.60]	94.67 ± 0.39	[94.18,95.16]
	FLIFuse	91.40 ± 0.38	[90.93,91.87]	76.73 ± 2.90	[73.14,80.33]	80.30 ± 2.17	[77.60,82.99]	78.11 ± 2.73	[74.73,81.50]	95.20 ± 0.16	[95.00,95.40]
COVID-19	HiFuse	87.30 ± 0.28	[86.96,87.64]	77.48 ± 0.74	[76.57,78.40]	82.22 ± 0.87	[81.13,83.30]	78.91 ± 0.82	[77.89,79.92]	92.99 ± 0.14	[92.82,93.17]
	FLIFuse	88.02 ± 0.39	[87.54,88.50]	78.57 ± 0.47	[77.98,79.15]	83.98 ± 0.98	[82.76,85.20]	80.20 ± 0.49	[79.60,80.81]	93.36 ± 0.26	[93.04,93.69]
Nerthus	HiFuse	93.28 ± 1.37	[91.58,94.99]	81.69 ± 2.64	[78.41,84.98]	79.96 ± 4.60	[74.25,85.67]	80.62 ± 3.53	[76.25,85.00]	96.00 ± 0.64	[95.20,96.79]
	FLIFuse	94.34 ± 0.89	[93.24,95.44]	85.91 ± 2.80	[82.44,89.38]	83.04 ± 3.09	[79.20,86.87]	84.21 ± 2.41	[81.22,87.20]	96.63 ± 0.53	[95.98,97.28]

On this basis, we further conducted the same cross-validation on the Nerthus endoscopic dataset to examine the model’s performance in cross-domain scenarios. The results showed that FLIFuse achieved an F1 score of 84.21%, representing an improvement of approximately 3.6 percentage points compared with HiFuse. Its accuracy was 94.34%, with recall and specificity of 85.91% and 96.63%, respectively. All mean values were also higher than those of HiFuse. Although the confidence intervals of some metrics overlapped, the direction of performance improvement was relatively consistent. Taken together, the results across the three datasets indicate that the performance advantage of FLIFuse over HiFuse has good reproducibility and stability under different data splits, which can provide more sufficient evidence for the robustness of the model.

### 3.8 Ablation experiment

This study aims to comprehensively evaluate the specific impact of each component within the FLIFuse model. This is achieved through a series of detailed ablation experiments conducted on the classification dataset containing normal, bacterial pneumonia, and viral pneumonia. Throughout the experiments, we strictly adhere to the principle of the control variable method, ensuring that all settings remain highly consistent except for the model component under test. This approach enables precise measurement of each components contribution to model performance.

To systematically validate the effectiveness of the three-branch architecture of the FLIFuse model, this study carefully designed ablation experiments. Specifically, we constructed a simplified model containing only the local feature extraction branch and another simplified model containing only the global feature extraction branch. Subsequently, a detailed comparative analysis was conducted between the classification performance of these two simplified models on the ChestXRay2017 dataset and that of the complete three-branch FLIFuse model, the relevant experimental results are presented in **Table 5**. By comparing changes in classification accuracy under different branch configurations, we can clearly identify the specific roles of each branch in enhancing the model’s discriminative power, thereby validating the necessity of the three-branch parallel design and providing a basis for making reasonable trade-offs between performance and efficiency.

The data in **Table 5** clearly demonstrate that the FLIFuse model achieves a significant improvement in accuracy, reaching 90.5% compared to 89.0% for the local feature extraction branch and 89.4% for the global feature extraction branch. Regarding recall, both the FLIFuse model and the global feature extraction branch achieve 73.4%, higher than the 71.4% of the local feature extraction branch. Regarding precision, the FLIFuse model achieves 68.9%, outperforming both the local feature extraction branch 66.1% and the global feature extraction branch 68.3%. The F1 score for the FLIFuse model 70.8%, also higher than the local feature extraction branch at 68.3% and the global feature extraction branch at 70.4%. These data suggest to some extentthat the three-branch architecture of FLIFuse exhibits better overall performance in the classification task on the ChestXRay2017 dataset. This indicates that local and global features capture complimentary discriminative information and the fusion branch can integrate the advantages of both, thereby improving the overall classification capability of the model.

**Table 5:** Three-branch architecture ablation experiment.

Methods	Acc (%)	Re (%)	Pre (%)	F1 (%)
Local Feature Path	89.0	71.4	66.1	68.3
Global Feature Path	89.4	73.4	68.3	70.4
FLIFuse	90.5	73.4	68.9	70.8

To systematically validate the effectiveness of the ExtraDW module introduced in the FLIFuse method, this study employs a controlled variable approach. Specifically, the original ExtraDW module in FLIFuse was sequentially replaced with the ResNet base block (Chowdhury et al., 2020), RepVGG block (Pogorelov et al., 2017), PConv block (He et al., 2016), MobileOne block (Ding et al., 2021), and GhostNetV3 module (Chen et al., 2023) to construct a series of comparative models for experimentation. Detailed experimental results are presented in **Table 6**. The ExtraDW module performs best across all metrics, with an accuracy of 91.4% and an F1 score exceeding 72%, while also maintaining high precision and recall rates. In contrast, modules such as the ResNet module and RepVGG module exhibit significantly lower performance across all metrics. For example, the ResNet module achieves an accuracy of only 84.5% and an F1 score of 61.5%. The results of this ablation experiment directly demonstratethe differences in actual performance among the candidate modules in the pneumonia classification task. They provide experimental evidence supporting the selection of the ExtraDW module as the local feature extraction component. The ExtraDW module seperates spatial feature extraction from channel fusion through depthwise seperable convolution, reducing computational cost while preserving detailed information, thereby achieving more accurate classification under resource-constrained conditions.

**Table 6:** Local feature extraction branch ablation experiment.

Methods	Acc (%)	Re (%)	Pre (%)	F1 (%)
ResNet block	84.5	66.2	58.4	61.5
RepVGG block	86.7	60.2	66.9	63.0
PConv block	89.0	68.8	74.3	71.1
MobileOne block	87.8	64.2	70.3	66.7
GhostNetV3 block	90.5	70.9	75.7	72.9
ExtraDW block	91.4	72.7	76.8	74.4

To validate the effectiveness of the improved Conv2Next block in the global feature extraction branch of FLIFuse, this study replaced the original Conv2Next block with ViT block, Swim Transformer block, and ConvNext modules to build comparative models and conduct experiments. Experimental results are detailed in **Table 7**. Combined with the specific metric data, it is evident that the Conv2Next block achieves the best performance. that the Conv2Next block possesses significant advantages in multi-dimensional performance and is a better choice for enhancing theoverall performance of the model. The Conv2Next block enlarges the receptive field at relatively low computational cost through large-kernel depthwise convolution, thereby enhancing global semantic modeling capability, which makes it perform better than other modules in the pneumonia classification tasks.

**Table 7:** Global feature extraction branch ablation experiment.

Methods	Acc (%)	Re (%)	Pre (%)	F1 (%)
ViT block	90.4	70.2	74.8	72.1
Swin Transforemer block	90.9	72.4	76.7	74.1
ConvNext block	91.0	70.5	74.8	72.3
Conv2Next block	91.4	72.7	76.8	74.4

## 4 Discussion

The FLIFuse model proposed in this study achieved an accuracy of 91.4% in the task of classifying bacterial and viral pneumonia from chest X-ray images, while keeping the number of parameters and computational cost at 33.428 M and 6.612 GFLOPs, respectively. Compared with models such as HiFuse, ConvNext, EfficientNetV2, and ViT, FLIFuse significantly reduces the demand for hardware resources while maintaining high classification accuracy, giving it certain deployment potential in resources-contrained scenario.

From an architectural perspective, FLIFuse employed a three-branch parallel structure that handled local details, global semantics, and multiscale fusion, respectively. This design enabled the model to capture both the overall characteristics of X-ray images—such as diffuse interstitial changes—and local lesion features, such as ground-glass opacities and small nodules. Specifically, the ExtraDW module in the local branch enhanced sensitivity to subtle lesions at a relatively low computational cost; the Conv2Next module in the global branch expanded the receptive field while keeping the number of parameters under control; and the FLI Block achieved information complementarity between global context and local details through the synergy of channel and spatial attention. The results of the ablation experiments further demonstrated that the aforementioned branches and modules all make positive contributions to the final classification performance, removing any single component leads to a decrease in accuracy, thereby validating the rationality of the three-branch architecture.

In terms of generalization ability, FLIFuse achieved an F1 score of 85.8% on the Nerthus intestinal cleanliness assessment dataset, outperforming models such as HiFuse, ViT, and Swin Transformer. This result suggests that the visual features extracted by this model exhibit certain task-independence and have the potential to be extended from chest X-rays to other medical image analysis scenarios. The stability of this transfer performance still needs to be further validated across more diverse datasets and real-world data.

It should be noted that this study still has room for further improvement in the following aspects. First, the data used in the experiments are publicly available retrospective datasets, and the sample sources and acquisition conditions are relatively concentrated. In the future, incorporating data from more sources and at a larger scale will help further validate the generalization ability of the model. Second, the current study is mainly evaluated on single-center data, and multicenter external validation can be conducted in subsequent work to more comprehensively examine the stability of the model, under different devices and acquisition protocols. In terms of statistical evaluation, this paper has conducted repeated experiments on key models and reported confidence intervals, and similar analyses can be extended to more models in the future to enhance statistical robustness. Regarding interpretability analysis this paper has qualitatively demonstrated the regions of interest using Grad-CAM, and more systematic interpretability methods can be combined in future work for further investigation. Overall, the current work mainly focuses on computational model evaluation, and prospective clinical validation as well as engineering issues in practical deployment will serve as important directions for subsequent research.

Based on the above analysis, future work could focus on the following areas: first further evaluating the model’s robustness on clinical image data from multiple sources; second, leveraging the model’s lightweight nature to explore deployment solutions and inference acceleration strategies mobile or embedded devices; and third, further investigating the transferability of this architecture to other diseases and different imaging modalities, so as to continue advancing its validation and application in clinical auxiliary diagnosis.

## 5 Conclusions

This study proposes FLIFuse, a lightweight, high-precision medical image classification model designed to assist in the differential diagnosis of bacterial and viral pneumonia based on X-ray images. The model employs an innovative three-branch parallel architecture: the ExtraDW module extracts local detailed features; the Conv2Next module models global semantic information; and the FLI Block achieves multi-scale fusion of local and global features, thereby enhancing feature representation capabilities while keeping computational overhead in check. On the ChestXRay2017 dataset, FLIFuse achieved a classification accuracy of 91.4%, with 33.428 M parameters and 6.612 GFLOPs of computational cost, striking a good balance between accuracy and efficiency, and demonstrating potential application value in computationally constrained scenarios. Cross-domain adaptability experiments on the COVID-19 chest X-ray database and the Nerthus dataset further demonstrated that the model possesses certain cross-disease and cross-modality transferability. Ablation experiments validated the positive contribution of the three-branch architecture and each core module to classification performance. Overall, FLIFuse provides a technical solution that balances accuracy and efficiency for the radiological identification of pneumonia pathogens. Future work could focus on validation using multicenter clinical data, optimizing model deployment on mobile or embedded devices, and exploring transferable applications across a broader range of medical imaging tasks.

## Data Availability

The datasets presented in this study can be found in online repositories. The names of the repository/repositories and accession number(s) can be found in the article/supplementary material.
